# Comparisons of Quality, Correctness, and Similarity Between ChatGPT-Generated and Human-Written Abstracts for Basic Research: Cross-Sectional Study

**DOI:** 10.2196/51229

**Published:** 2023-12-25

**Authors:** Shu-Li Cheng, Shih-Jen Tsai, Ya-Mei Bai, Chih-Hung Ko, Chih-Wei Hsu, Fu-Chi Yang, Chia-Kuang Tsai, Yu-Kang Tu, Szu-Nian Yang, Ping-Tao Tseng, Tien-Wei Hsu, Chih-Sung Liang, Kuan-Pin Su

**Affiliations:** 1 Department of Nursing Mackay Medical College Taipei Taiwan; 2 Department of Psychiatry Taipei Veterans General Hospital Taipei Taiwan; 3 Division of Psychiatry, School of Medicine, National Yang-Ming University Taipei Taiwan; 4 Department of Psychiatry Kaohsiung Medical University Hospital Kaohsiung Taiwan; 5 Department of Psychiatry College of Medicine Kaohsiung Medical University Kaohsiung Taiwan; 6 Department of Psychiatry Kaohsiung Municipal Siaogang Hospital Kaohsiung Medical University Kaohsiung Taiwan; 7 Department of Psychiatry Kaohsiung Chang Gung Memorial Hospital Kaohsiung Taiwan; 8 Department of Neurology Tri-Service General Hospital National Defense Medical Center Taipei Taiwan; 9 Institute of Epidemiology and Preventive Medicine College of Public Health National Taiwan University Taipei Taiwan; 10 Department of Dentistry National Taiwan University Hospital Taipei Taiwan; 11 Department of Psychiatry Tri-service Hospital, Beitou branch Taipei Taiwan; 12 Department of Psychiatry Armed Forces Taoyuan General Hospital Taoyuan Taiwan; 13 Graduate Institute of Health and Welfare Policy National Yang Ming Chiao Tung University Taipei Taiwan; 14 Institute of Biomedical Sciences Institute of Precision Medicine National Sun Yat-sen University Kaohsiung Taiwan; 15 Department of Psychology College of Medical and Health Science Asia University Taichung Taiwan; 16 Prospect Clinic for Otorhinolaryngology and Neurology Kaohsiung Taiwan; 17 Department of Psychiatry E-Da Dachang Hospital I-Shou University Kaohsiung Taiwan; 18 Department of Psychiatry E-Da Hospital I-Shou University Kaohsiung Taiwan; 19 Department of Psychiatry Tri-service Hospital Beitou branch Taipei Taiwan; 20 Department of Psychiatry National Defense Medical Center Taipei Taiwan; 21 College of Medicine China Medical University Taichung Taiwan; 22 Mind-Body Interface Laboratory China Medical University and Hospital Taichung Taiwan; 23 An-Nan Hospital China Medical University Tainan Taiwan

**Keywords:** ChatGPT, abstract, AI-generated scientific content, plagiarism, artificial intelligence, NLP, natural language processing, LLM, language model, language models, text, textual, generation, generative, extract, extraction, scientific research, academic research, publication, publications, abstracts

## Abstract

**Background:**

ChatGPT may act as a research assistant to help organize the direction of thinking and summarize research findings. However, few studies have examined the quality, similarity (abstracts being similar to the original one), and accuracy of the abstracts generated by ChatGPT when researchers provide full-text basic research papers.

**Objective:**

We aimed to assess the applicability of an artificial intelligence (AI) model in generating abstracts for basic preclinical research.

**Methods:**

We selected 30 basic research papers from Nature, Genome Biology, and Biological Psychiatry. Excluding abstracts, we inputted the full text into ChatPDF, an application of a language model based on ChatGPT, and we prompted it to generate abstracts with the same style as used in the original papers. A total of 8 experts were invited to evaluate the quality of these abstracts (based on a Likert scale of 0-10) and identify which abstracts were generated by ChatPDF, using a blind approach. These abstracts were also evaluated for their similarity to the original abstracts and the accuracy of the AI content.

**Results:**

The quality of ChatGPT-generated abstracts was lower than that of the actual abstracts (10-point Likert scale: mean 4.72, SD 2.09 vs mean 8.09, SD 1.03; *P*<.001). The difference in quality was significant in the unstructured format (mean difference –4.33; 95% CI –4.79 to –3.86; *P*<.001) but minimal in the 4-subheading structured format (mean difference –2.33; 95% CI –2.79 to –1.86). Among the 30 ChatGPT-generated abstracts, 3 showed wrong conclusions, and 10 were identified as AI content. The mean percentage of similarity between the original and the generated abstracts was not high (2.10%-4.40%). The blinded reviewers achieved a 93% (224/240) accuracy rate in guessing which abstracts were written using ChatGPT.

**Conclusions:**

Using ChatGPT to generate a scientific abstract may not lead to issues of similarity when using real full texts written by humans. However, the quality of the ChatGPT-generated abstracts was suboptimal, and their accuracy was not 100%.

## Introduction

ChatGPT is an advanced language model that marks a significant milestone in the development of conversational artificial intelligence (AI) systems, highlighting its contribution to the field of natural language processing. In scientific writing, ChatGPT may provide valuable support for drafting manuscripts, summarizing articles, translating languages, and refining text structures or wording [1]. Although the use of ChatGPT as a research assistant for scientific paper composition and publication has gained significant attention, several concerns have emerged. First, AI models trained on vast amounts of text data may inadvertently generate content that closely resembles existing scientific work, leading to instances of plagiarism [1]. Second, AI models can generate seemingly plausible content that lacks accuracy or may unintentionally introduce errors. Moreover, as these models are trained on data sets that may contain biases, they can inadvertently amplify such biases without proper supervision by domain experts. This raises concerns about the trustworthiness and reliability of the generated scientific papers. Third, as AI capabilities progress, there is concern that AI-generated scientific papers could deceive reviewers and educators, potentially resulting in unwarranted acceptance or recognition [2].

A recent study investigated the capabilities of AI language models in generating scientific abstracts. In this study, we provided the “title” and “journal style” to ChatGPT and prompted it to generate a scientific abstract [3]. We found that the abstracts generated by ChatGPT exhibited a high level of fluency and successfully deceived human reviewers. However, these abstracts contained fabricated data and high percentages of plagiarism and AI-generated content. Another study provided the full text of psychiatric papers (excluding the original abstract) to an AI model and prompted it to generate an abstract with the same style (structured or unstructured) as the original paper [4]. Notably, the structured abstracts generated by ChatGPT exhibited comparable quality to real abstracts, whereas the unstructured abstracts generated by ChatGPT displayed lower quality than the real abstracts [4]. Plagiarism was low in the AI-generated abstracts (7.55%). However, 30% (6/20) of the conclusions drafted by AI were incorrect [4]. Collectively, the observed disparities in the plagiarism percentages between these two studies can be attributed to the distinct prompt instructions used. Additionally, both studies used clinical medical research articles as the basis for generating scientific abstracts using AI models.

In this study, we aimed to assess the applicability of an AI model in generating abstracts for basic preclinical research. Drafting abstracts of basic studies may be more challenging than those of clinical studies because basic research delves into fundamental scientific principles, mechanisms, and theoretical concepts that can be highly complex and specialized. Basic research papers primarily target experts, scientists, and researchers with a high level of domain-specific knowledge. To date, no study has investigated the quality of abstracts generated by ChatGPT using full-text prompts. We hypothesized that the quality of abstracts generated by ChatGPT would differ from the quality observed in real abstracts.

## Methods

### Overview

We evaluated the quality and accuracy of the abstracts of basic research papers generated by ChatGPT. Basic research abstracts can be classified as unstructured or structured, and structured abstracts may consist of 3 or 4 subheadings. We selected 10 papers published in *Nature*, 10 in *Genome Biology*, and 10 in *Biological Psychiatry*, each. The abstract formats in *Nature*, *Genome Biology*, and *Biological Psychiatry* were unstructured, structured with 3 subheadings, and structured with 4 subheadings.

### Generation of Abstract

We used ChatPDF [5], which is based on ChatGPT 3.5, to efficiently analyze the PDF file content. ChatPDF offers users a summary and answers questions regarding PDF files without nonexistent or self-created content, which is a major concern in ChatGPT [6]. We provided ChatPDF with full texts of the selected papers after excluding abstracts. The prompts provided to the ChatPDF were the following: “Please summarize a 200-word abstract” for papers published in *Nature*; “Please summarize a 250-word abstract including 3 paragraphs, namely ‘Background,’ ‘Results,’ and ‘Conclusions,’ for papers published in *Genome Biology*, and “Please summarize a 250-word abstract including 4 paragraphs, namely ‘Background,’ ‘Methods,’ ‘Results,’ and ‘Conclusions,’ for papers published in *Biological Psychiatry*.

### Similarity, AI Content, and Subheadings

We evaluated similarity using a plagiarism comparison platform [7], which compares the generated and original abstracts for duplicate content. We also examined the AI output detectors for both generated and original abstracts using GPTZero [8]. GPTZero determined the content either as “entirely by AI,” “parts by AI,” or “entirely by a human” in each original and generated abstract. GPTZero also uses 2 measures to determine whether a text has been written by a human: perplexity and burstiness. Perplexity is a measure that assesses how well a language model predicts a text sample. A lower perplexity suggests that the text is easy to predict, which may indicate that it sounds more like machine-generated text. Burstiness is the occurrence of uncommon items appearing in random clusters over time (ie, creative variability), which is based on the idea that humans tend to mix long and short sentences, whereas AI sentences are uniform. Therefore, low burstiness suggests machine-generated text. We also compared the word counts in each subheading.

### Experts and Quality Evaluation

A total of 8 experts in scientific writing and publishing evaluated the quality of the abstracts after reading the full texts of the research papers. The quality score was assigned using a Likert scale ranging from 0 to 10 (worst=0, not bad=5, extremely good=10) while considering the concise, precise, functional, unbiased, comprehensive, and self-sufficient aspects of the abstracts. In addition, experts were asked to identify abstracts written by human authors. Finally, the experts validated the conclusions generated by ChatPDF during the unblinding phase.

### Statistical Analyses

Statistical analyses were performed using the SPSS (version 26; SPSS Inc). Scatter plots were generated using GraphPad Prism (version 8; GraphPad Software Inc). The pie charts were generated using Microsoft Excel 365. Box and distribution plots were generated in R using the ggplot2 package.

Group differences were analyzed using 2-sample *t* tests (for continuous variables) and chi-square tests (for categorical variables). If more than 20% of the cells expected cell counts of less than 5, the Fisher exact test was conducted for categorical variables. Correlations between 2 variables were examined using the Pearson correlation coefficient. Linear regression analysis was performed to determine the predictors of the scores. We considered the following predictors: the h-index and research year of the expert (continuous variables), the format of the abstract (categorical variable), the publication date of the paper (continuous variable), and whether the paper was open access (categorical variable). All *t* test analyses were 2-tailed, and *P*<.05 was considered significant.

## Results

### Characteristics of the Experts and Included Articles

**Figure 1:**
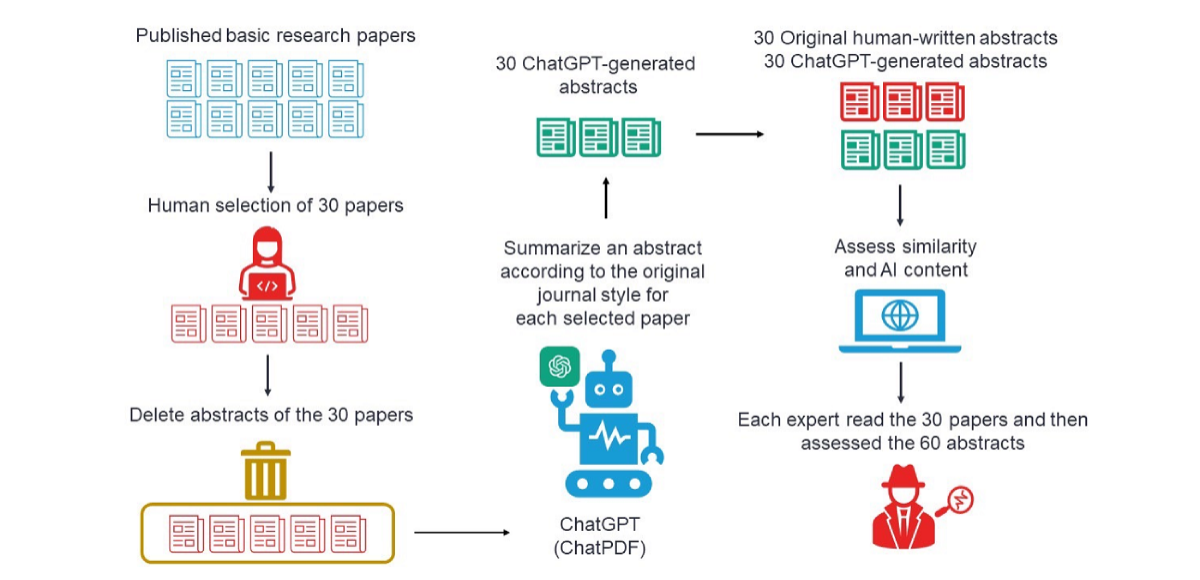
The visual representation of the operational process. AI: artificial intelligence.

The h-indices of the 8 experts ranged from 25 to 56, and their research experience ranged from 16 to 29 years (Table S1 in Multimedia Appendix 1). The 30 selected articles were published between the years 2022 and 2023. The average word count of the abstracts (Table S2 in Multimedia Appendix 1) was 240.30 (SD 21.37) for *Biological Psychiatry*, 212.30 (SD 20.66) for *Genome Biology*, and 142.90 (SD 11.43) for *Nature*. The full texts of the generated abstracts are listed in Table S3 in Multimedia Appendix 1. The visual representation of the operational process is presented in Figure 1. The details of the operational processes are presented in Multimedia Appendix 1.

### Similarity

**Figure 2:**
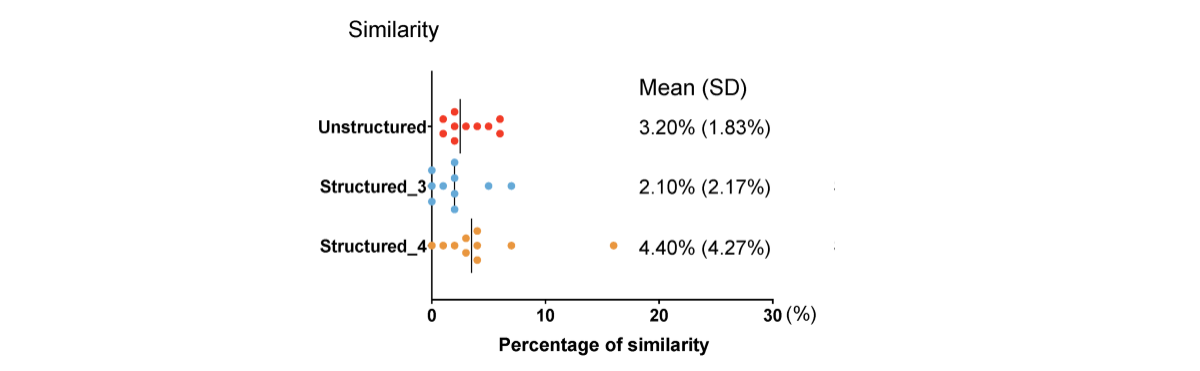
Similarity (duplicate content) of the ChatGPT-generated abstracts (unstructured abstracts, abstracts with 3 subheadings, and abstracts with 4 subheadings).

The mean similarity (duplicate content) between the original and the generated abstracts was 3.20% (SD 1.83%) for unstructured abstracts, 2.10% (SD 2.17%) for structured abstracts with 3 subheadings, and 4.40% (SD 4.27%) for structured abstracts with 4 subheadings (Figure 2).

### AI Content

**Figure 3:**
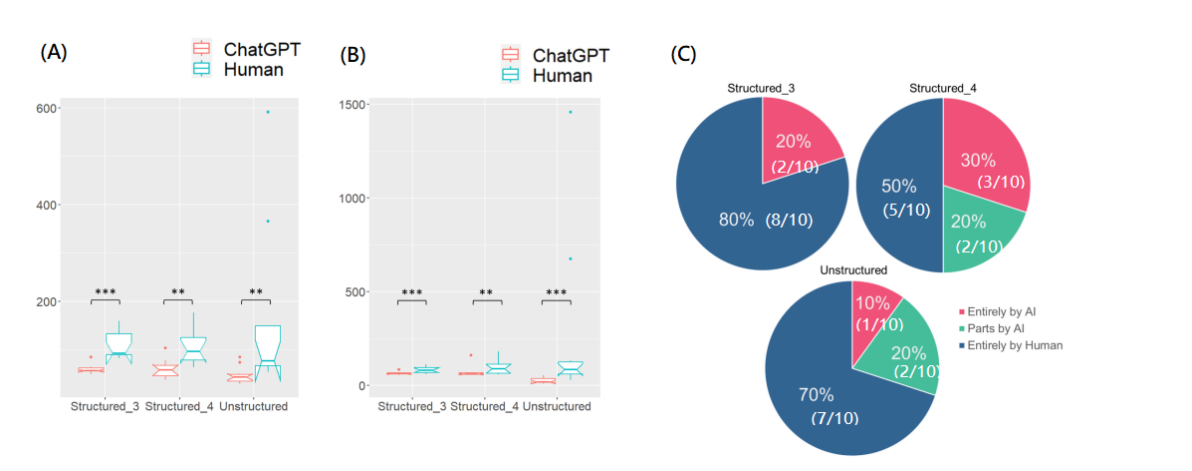
Artificial intelligence (AI) content of the ChatGPT-generated and the original author-written abstracts concerning (A) perplexity, (B) burstiness, and (C) judgement (unstructured abstracts, abstracts with 3 subheadings, and abstracts with 4 subheadings). *P<.05, **P<.01, ***P<.001.

In the AI content analysis, the original abstracts had significantly higher perplexity scores than generated abstracts (structured abstracts with 3 subheadings: mean 110.72, SD 29.97 vs mean 59.49, SD 10.01; *P*<.001; structured abstracts with 4 subheadings: mean 105.17, SD 36.45 vs mean 60.58, SD 19.58; *P*=.002; unstructured abstracts: mean 165.57, SD 55.89 vs 47.70, SD 18.15; *P*=.002; Figure3A and Tables S4 and S5 in Multimedia Appendix 1). The original abstracts also had significantly higher burstiness scores compared to the generated abstracts (structured abstracts with 3 subheadings: mean 83.14, SD 17.15 vs mean 64.31, SD 8.24; *P*=.009; structured abstracts with 4 subheadings: mean 94.95, SD 38.38 vs mean 70.36, SD 31.48; *P*=.015; unstructured abstracts: mean 271.13, SD 459.07 vs mean 24.90, SD 15.49; *P*<.001; Figure 3B and Table S4 and S5 in Multimedia Appendix 1). Among the 30 AI-generated abstracts, 20 were judged as entirely human-generated content, 4 as part of the AI content, and 6 as entirely AI-generated content (Figure 3C and Table S4).

### Quality, Correlation, and Predictors

**Figure 4:**
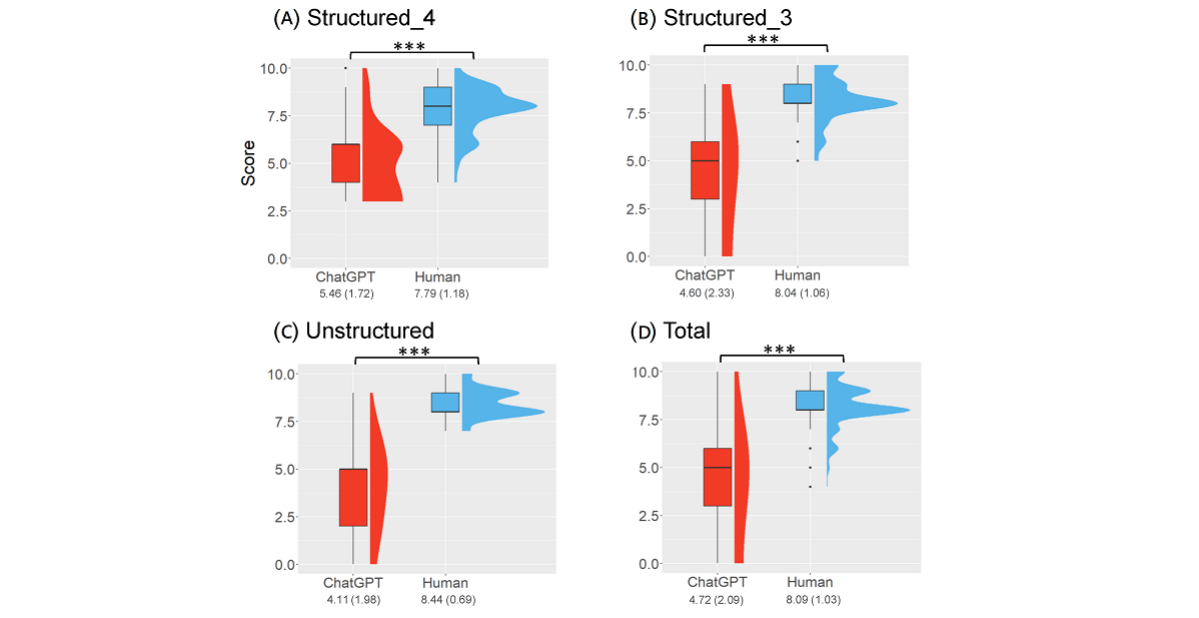
Comparisons of the quality of the ChatGPT-generated and the original author-written abstracts. (A) abstracts with 3 subheadings; (B) abstracts with 4 subheadings; (C) unstructured abstracts; (D) all abstracts. Values on the x-axis are reported as mean (SD). ***P<.001.

The original abstracts were assessed to have significantly higher quality than AI-generated abstracts (mean 8.09, SD 1.03 vs mean 4.72, SD 2.09; Figure 4D and Table 1). Comparison of various formats is as follows: unstructured abstracts (8.44, SD 0.69 vs mean 4.11, SD 1.98; Figure 4C), structured abstracts with 3 subheadings (mean 8.04, SD 1.06 vs mean 4.60, SD 2.33; Figure 4B), and structured abstracts with 4 subheadings (mean 7.79, SD 1.18 vs mean 5.46, SD 1.72; Figure 4A). The quality score was negatively correlated with the original and AI-generated abstracts in structured abstracts with 3 subheadings (coefficient –0.37; *P*<.01) and structured abstracts with 4 subheadings (coefficient –0.32; *P*<.01) but not in unstructured abstracts (*P*=.08; Table S6 in Multimedia Appendix 1). Table S7 in Multimedia Appendix 1 lists the predictors of abstract quality scores. A higher h-index of blinded raters (*P*<.001), more senior raters (*P*=.04), and structures with 4 subheadings (*P*=.03) were positively correlated with the quality score of AI-generated abstracts, while unstructured abstracts were negatively correlated (*P*=.003). Regarding human-written abstracts, a higher h-index of blinded raters (*P*<.001) and unstructured abstracts (*P*=.002) were positive predictors, whereas senior raters and those abstracts structured with 4 subheadings were negative predictors.

**Table 1:** Quality of ChatGPT-generated abstracts versus human-written abstracts.

Abstract format	Abstracts written by ChatGPT, mean (SD)	Abstracts written by humans, mean (SD)	Difference (95% CI)	*P* value
Structured with 4 subheadings (n=160)	5.46 (1.72)	7.79 (1.18)	–2.33 (–2.79 to –1.86)	<.001
Structured with 3 subheadings (n=160)	4.60 (2.33)	8.04 (1.06)	–3.44 (–4.00 to –2.87)	<.001
Unstructured (n=160)	4.11 (1.98)	8.44 (0.69)	–4.33 (–4.79 to –3.86)	<.001
All formats (N=480)	4.72 (2.09)	8.09 (1.03)	–3.36 (–3.66 to –3.07)	<.001

### Word Counts in Each Subheading

In structured abstracts with 4 subheadings, AI-generated abstracts had more words in the “conclusions” section compared to abstracts written by humans (71.7 vs 40.9 words; *P*=.002), but fewer words were used in the “results” section (58.8 vs 85.2 words; *P*=.001; Table 2). In structured abstracts with 3 subheadings, AI generated more words in the “conclusions” section compared to humans (92.8 vs 40.9 words; *P*<.001), but it generated fewer words in the “results” section (66.2 vs 135.6 words; *P*<.001; Table 2).

**Table 2:** Word counts for ChatGPT-generated abstracts versus human-written abstracts.

Abstract		ChatGPT (mean, SD)	Human (mean, SD)	Difference (95% CI)	*P* value
**Structured abstract (4 subheadings)**					
	Background	59.70 (9.81)	65.20 (20.07)	–5.50 (–20.76 to 9.76)	.45
	Methods	49.20 (8.63)	60.00 (30.87)	–10.80 (–33.39 to 11.79)	.31
	Results	58.80 (15.39)	85.20 (15.02)	–26.40 (–40.75 to –12.05)	.001
	Conclusions	71.70 (15.16)	40.90 (20.62)	30.80 (13.65 to 47.95)	.002
	Total words	239.40 (21.76)	251.30 (24.94)	–11.90 (–33.98 to 10.18)	.27
**Structured abstract (3 subheadings)**					
	Background	53.30 (16.06)	50.50 (14.06)	2.80 (–11.44 to 17.04)	.68
	Results	66.20 (10.42)	135.60 (33.47)	–69.40 (–94.10 to –44.70)	<.001
	Conclusions	92.80 (12.73)	34.90 (13.03)	–64.90 (–96.94 to –32.86)	<.001
	Total words	212.30 (21.78)	221.00 (28.43)	57.90 (45.75 to 70.05)	.45
Unstructured abstract (total words)		142.90 (12.05)	207.60 (36.27)	–64.70 (–91.63 to –37.77)	<.001

### Accuracy of Authors’ Judgement and Correctness of Conclusions

Table 3 shows the accuracy of the judgments of the 8 experts. The judgment accuracies were 83.75% (67/80) for structured abstracts with 4 subheadings, 96.28% (77/80) for structured abstracts with 3 subheadings, and 100% (80/80) for unstructured abstracts. Finally, among the 30 ChatGPT-generated abstracts, 3 showed wrong conclusions.

**Table 3:** Accuracy of the judgement of abstracts generated by humans or ChatGPT.

Abstract	Accuracy, %	Proportion, n/N
Structured abstract with 4 subheadings	83.75	67/80
Structured abstract with 3 subheadings	96.28	77/80
Unstructured abstract	100	80/80

## Discussion

### Principal Findings

In this study, we prompted ChatGPT with full texts of basic research papers and requested that abstracts be generated according to the style guidelines of the journals. The main findings are as follows: first, the similarity levels of the AI-generated abstracts were not particularly high (less than 20%) when using full texts as prompts. Second, in AI content analysis, the AI content detector tool can only identify one-third of the AI-generated abstracts. However, when compared to the original abstracts, AI-generated abstracts were rated as having lower scores for perplexity and burstiness, indicating that the difference between AI and human writing could still be detected. Third, in terms of quality rating, the AI-generated abstracts scored significantly lower than the original abstracts, and the difference was substantial (3.36 points on a 10-point rating scale). Fourth, the AI and human writers demonstrated different tendencies in the allocation of word counts to subheadings. Human writers tended to use more words in the results section, whereas AI writers tended to allocate a larger proportion of words to the conclusion section. Fifth, the identification accuracy between AI-generated and human-written abstracts was high (93%). In particular, 100% of the accuracy was achieved using the unstructured style. Finally, 3 of the 30 AI-generated abstracts yielded incorrect conclusions.

AI-generated content may exhibit high perplexity and burstiness scores. Perplexity and burstiness are metrics related to randomness and chaos, respectively. Perplexity measures the level of randomness or complexity in word usage, whereas burstiness quantifies variability in sentence length, structure, and tempos [9]. In natural language processing, AI models tend to write with a consistent tempo, resulting in low perplexity and burstiness, whereas human writers often exhibit bursts and lulls in their writing styles [9]. Although the AI content detector we used did not demonstrate sufficient accuracy in detecting AI-generated content, we still observed a significant difference in perplexity and burstiness between the human-written and AI-generated abstracts. It has been suggested that an AI output detector is better at distinguishing between original and ChatGPT-generated articles than a plagiarism detector [10].

In terms of quality assessment, our findings revealed that ChatGPT performed best in generating structured abstracts with 4 subheadings, followed by structured abstracts with 3 subheadings and unstructured abstracts. It appears that AI could generate higher-quality content when provided with more instructions in the form of subheadings, particularly when the word count per subheading is limited, as per our prompts. Notably, in unstructured abstracts, AI tended to focus more on introducing the study and its conclusion, while allocating less attention to the methods and results sections, which are crucial components presenting the core knowledge of the study. This observation is consistent with the distribution of words in structured abstracts. Human writers tended to allocate more words to the results section, whereas AI emphasized the conclusion section. This phenomenon contributes to the shallowness of the content generated by AI. The finding of higher-quality structured abstracts than unstructured abstracts generated by AI aligns with that of our previous study [4]. However, the difference in quality was more pronounced in this study involving preclinical research papers compared to clinical papers used in our previous study. ChatGPT appears to face greater challenges in interpreting professional preclinical research papers, resulting in lower quality and shallower abstracts for this specific domain. Nonetheless, a previous study [11] successfully used ChatGPT to generate highly convincing fraudulent scientific papers on neurosurgery, accomplished by fabricated data and tables using careful and step-by-step prompts over a duration of approximately 1 hour. Although some mistakes were identified in the generated articles, this finding suggests that AI-generated content can be concise and intricately crafted using multiple instructions (prompts) or training.

In contrast to previous studies [3,4], the accuracy of identifying AI-generated abstracts was only 68% in Gao et al [3] and 78% in our previous study [4]. However, the accuracy of identification by the 8 experts was notably high, reaching 93% for all abstracts and 100% for unstructured abstracts. The challenges associated with comprehending the intricate knowledge of preclinical research, which often leads to lower-quality outputs, contribute to experts’ ability to differentiate AI-generated content. The accuracy of identification demonstrated a correlation with the quality score—a higher identification rate was associated with lower quality. Notably, experts were able to identify 100% of the AI-generated unstructured abstracts, which scored lower in quality compared to structured abstracts. Finally, not all abstracts generated by AI showed correct conclusions.

### Study Limitations

This study has several limitations. First, we used ChatPDF, a tool based on the ChatGPT 3.5. Therefore, the generalizability of our findings to other AI language models may be limited. Our results should be interpreted cautiously in the context of the current version of ChatPDF. Second, we provided only a single prompt for the AI model without any training. The results may differ when multiple prompts are used or when training procedures are incorporated. Third, we used a limited selection of AI content detectors. The applicability of our AI content detection results could vary if other tools were used. Finally, our study focused on generating scientific abstracts by prompting an AI model with scientific articles. However, there are numerous other applications in scientific research. Further investigation is necessary to evaluate the potential of AI in scientific writing.

### Conclusions

In conclusion, AI-generated content can be identified by both human experts and computer-based analyses. AI may be capable of deceiving human experts in common or less specialized subjects. However, the limitations of AI have become more apparent in professional domains. AI-driven output detectors based on linguistic analyses are promising in computer science. Nevertheless, we anticipate ongoing competition between AI output detectors and writers.

## Appendix

Multimedia Appendix 1 Additional statistics and details of the operational process.

## Abbreviations

AI: artificial intelligence

## Data Availability

The data sets generated and analyzed during this study are available from the corresponding author upon reasonable request.
