# Supplementary material for: Comparisons of Quality, Correctness, and Similarity Between ChatGPT-Generated and Human-Written Abstracts for Basic Research: Cross-Sectional Study
Source: J Med Internet Res. 2023 Dec 25;25:e51229. doi: 10.2196/51229 (PMC10760418; doi:10.2196/51229)
Supplement: Multimedia Appendix 1 [file jmir_v25i1e51229_app1.docx]

Table S1. Characteristics of the eight experts in research and academic paper writing^a^

| Ratters | Research years | Numbers publications | H-index | Academic status |
| --- | --- | --- | --- | --- |
| Expert 1 | 16 | 196 | 26 | MD |
| Expert 2 | 18 | 110 | 25 | Prof |
| Expert 3 | 25 | 375 | 56 | Prof |
| Expert 4 | 22 | 143 | 31 | Prof |
| Expert 5 | 24 | 200 | 35 | Prof |
| Expert 6 | 29 | 460 | 54 | Prof |
| Expert 7 | 20 | 152 | 51 | Prof |
| Expert 8 | 15 | 122 | 28 | Prof |

^a^ The number of publications are according to search on PubMed up to April 12, 2023.

Table S2. Characteristics of the included articles

| Variable | Paper with structured abstract (4 subheadings) | Paper with structured abstract (3 subheadings) | Paper with unstructured abstract |
| --- | --- | --- | --- |
| Number | 10 | 10 | 10 |
| Range of year | 2022-2023 | 2022-2023 | 2022-2023 |
| Journals | Biological Psychiatry | Genome Biology | Nature |
| Open Assess | 7/10 | 10/10 | 5/10 |
| Word count of abstract | 240.30 (21.37) | 212.30 (20.66) | 142.90 (11.43) |
| Research type | Preclinical basic research | Preclinical basic research | Preclinical basic research |

Table S3. Abstracts generated by ChatGPT using ChatPDF

| Bio-Psy-1: Disruption of Astrocyte-Dependent Dopamine Control in the Developing Medial Prefrontal Cortex Leads to Excessive Grooming in Mice |
| --- |
| Background:  Compulsive repetitive behaviors are key features of many neuropsychiatric disorders, including obsessive-compulsive disorder (OCD) and Tourette syndrome. However, little is known about the neurobiology underlying the development of these behaviors at the cellular level or the neuromodulators involved. This study aimed to investigate the role of astrocyte-dependent dopamine control in the medial prefrontal cortex (mPFC) in compulsive grooming behavior in mice.  Methods:  We used a combination of optogenetics, electrophysiology, and behavioral assays to investigate the effects of astrocyte-specific disruption of dopamine signaling in the mPFC on grooming behavior in mice. We also examined changes in neuronal activity and synaptic plasticity in this brain region.  Results:  The study found that disruption of astrocyte-dependent dopamine control in the mPFC led to excessive grooming behavior in mice. This was accompanied by changes in neuronal activity and synaptic plasticity, suggesting that alterations in dopaminergic signaling can affect neural circuitry and behavior. We also found that optogenetic activation of the mPFC induced excessive grooming behavior, further supporting a role for this brain region in compulsive behaviors.  Conclusions:  This study provides new insights into the neurobiology underlying compulsive behaviors and highlights the importance of astrocyte-dependent dopamine control in the mPFC. The findings suggest that dysregulation of dopaminergic signaling may contribute to abnormal cortico-striato-thalamo-cortical loops implicated in OCD-like symptoms. The study also suggests that targeting astrocyte-dependent dopamine signaling may be a promising approach for developing new treatments for compulsive behaviors associated with neuropsychiatric disorders. However, further research is needed to fully understand how alterations in dopaminergic signaling affect neural circuitry and behavior and to develop effective therapies for these conditions. |
| Correctness of conclusion: correct |

| Bio-Psy-2: Spinophilin Limits Metabotropic Glutamate Receptor 5 Scaffolding to the Postsynaptic Density and Cell Type Speciﬁcally Mediates Excessive Grooming |
| --- |
| Background:  The sensorimotor striatum is a crucial basal ganglia input nucleus that integrates excitatory and modulatory inputs from diverse cortical and subcortical structures to promote the learning and execution of complex tasks. However, how endogenous spinophilin mediates striatal plasticity and SAPAP3- and mGluR5-dependent repetitive motor output is unknown. This study aimed to investigate the role of spinophilin in limiting metabotropic glutamate receptor 5 scaffolding to the postsynaptic density and mediating excessive grooming.  Methods:  Using a novel conditional spinophilin knockout (KO) mouse line, combined with behavioral, functional, biochemical, and proteomic approaches, we implicate spinophilin as a hub striatal signaling molecule that mediates MSN subtype-specific adaptations underlying repetitive motor output associated with increased mGluR5 function.  Results:  The results showed that spinophilin KO mice exhibited excessive grooming behavior that was rescued by selective re-expression of spinophilin in direct-pathway medium spiny neurons (dMSNs) but not indirect-pathway medium spiny neurons (iMSNs). We also found that spinophilin limits mGluR5 scaffolding to the postsynaptic density by regulating Homer1b/c binding to mGluR5. Furthermore, we identified several proteins whose expression was altered in the striatum of spinophilin KO mice.  Conclusions:  This study provides evidence for the critical role of spinophilin in regulating striatal plasticity and repetitive motor output associated with increased mGluR5 function. The findings suggest that targeting spinophilin or its downstream signaling pathways may be a promising therapeutic strategy for obsessive-compulsive spectrum disorders. Moreover, this research sheds light on the intricate workings of the sensorimotor striatum and how perturbations within it can lead to repetitive motor dysfunction in psychiatric disorders. |
| Correctness of conclusion: correct |

| Bio-Psy-3: Excessive Protein Accumulation and Impaired Autophagy in the Hippocampus of Angelman Syndrome Modeled in Mice |
| --- |
| Background:  Angelman Syndrome (AS) is a genetic neurodevelopmental disorder caused by defects of the 15q11.2-q13.1 chromosomal region, which is paternally imprinted in neurons. Most cases of AS have deletions or mutations of the gene encoding the ubiquitin-protein ligase E3A (UBE3A), a critical player of the ubiquitin-proteasome system, which is a protein degradation system involved in neuronal morphological maturation, synaptic plasticity, and cortical development.  Methods:  The study aimed to investigate the role of protein metabolism and autophagy in AS using mouse models. We performed CFC and immediate shock control paradigms, implantation of cannulae targeting the dHC of mice, bilateral injections of TAT-Beclin 1 and SBI-0206965 or vehicle, in vivo surface sensing of translation, RNA isolation followed by quantitative polymerase chain reaction analyses.  Results:  There was an increased rate of protein synthesis and impaired autophagy with a lack of stimulus-dependent dynamic regulation in AS mouse models. Autophagic flux was impaired in AS dHC, and there was a significant accumulation of autophagosomal structures, suggesting impaired protein degradation. The accumulation of immediate early gene (IEG) products in neurons affected brain function and led to neuropsychiatric symptoms in Angelman Syndrome.  Conclusions:  Altered protein metabolism due to an increased rate of protein synthesis and impaired autophagy with a lack of stimulus-dependent dynamic regulation is a critical biological alteration associated with AS. The dysfunction in the ubiquitin-proteasome system contributes to Angelman Syndrome's pathogenesis by impairing neuronal morphological maturation, synaptic plasticity, and cortical development. The findings suggest that targeting the protein degradation system or autophagy could be potential therapeutic targets for alleviating the symptoms associated with Angelman Syndrome. |
| Correctness of conclusion: correct |

| Bio-Psy-4: Excitatory Dysfunction Drives Network and Calcium Handling Deﬁcits in 16p11.2 Duplication Schizophrenia Induced Pluripotent Stem Cell– Derived Neurons |
| --- |
| Background:  Schizophrenia is a complex psychiatric disorder that is highly heritable, with 60% to 90% of risk attributed to genetic background. The 16p11.2 duplication is one of the most common genetic variants associated with schizophrenia. However, the molecular mechanisms underlying this association remain poorly understood.  Methods:  We used induced pluripotent stem cells (iPSCs) derived from patients with 16p11.2 duplication schizophrenia to investigate the cellular and molecular phenotypes associated with this genetic variant. We performed electrophysiological recordings, calcium imaging, and transcriptomic analyses to characterize the functional properties of iPSC-derived neurons.  Results:  iPSC-derived neurons from patients with 16p11.2 duplication schizophrenia exhibited excitatory dysfunction and calcium handling deficits compared to control neurons. Specifically, we observed reduced excitatory synaptic transmission, impaired calcium signaling, and altered expression of genes involved in calcium homeostasis and synaptic function.  Conclusions:  These findings suggest that the 16p11.2 duplication may contribute to the pathogenesis of schizophrenia by disrupting excitatory neurotransmission and calcium signaling in neurons. The use of iPSCs provides a powerful tool for studying the cellular and molecular mechanisms underlying complex psychiatric disorders like schizophrenia. Further research is needed to validate these findings in larger cohorts of patients and to explore potential therapeutic targets for this debilitating disorder. |
| Correctness of conclusion: correct |

| Bio-Psy-5: Histone Methyltransferase SETDB1 Regulates the Development of Cortical Htr3a-Positive Interneurons and Mood Behaviors |
| --- |
| Background:  GABAergic interneurons (INs) are critical for maintaining the balance between synaptic excitation and inhibition in the neocortex. Dysregulation of cortical INs is a potential pathogenesis for neuropsychiatric disorders. Histone methyltransferase SETDB1 plays a vital role in excitatory neurons and participates in cognition and mood behaviors, but no studies have reported its function in the GABAergic neuronal lineage.  Methods:  In this study, we investigated the role of SETDB1 in regulating the development of cortical Htr3a-positive interneurons and mood behaviors. We used immunobiology, luciferase assay, epigenomic editing, behavioral tests, RNA-seq, ATAC-seq, ChIP-seq, 3C, cell recording, and GE_NPC culture to perform their experiments.  Results:  SETDB1 regulates Htr3a transcription and the development of Htr3a1 INs. We also discovered that SETDB1 deficiency leads to decreased Htr3a expression and reduced 5-HT3R receptor-mediated currents in cortical INs. Furthermore, we observed that SETDB1-deficient mice exhibit anxiety- and depression-like behaviors.  Conclusions:  The results suggest that SETDB1 plays an important role in regulating the development of cortical Htr3a-positive interneurons and mood behaviors. Dysregulation of SETDB1 may contribute to neuropsychiatric disorders by altering GABAergic neurotransmission. The findings provide new insights into the molecular mechanisms underlying neuropsychiatric disorders and may lead to new therapeutic strategies for these conditions. Further research is needed to fully understand the complex interactions between histone modifications, gene expression, and behavior in the brain. |
| Correctness of conclusion: correct |

| Bio-Psy-6: CREB Binding at the Zfp189 Promoter Within Medium Spiny Neuron Subtypes Differentially Regulates Behavioral and Physiological Adaptations Over the Course of Cocaine Use |
| --- |
| Background:  Drug addiction is a complex disorder that involves persistent changes in the brain at the molecular, cellular, and circuit levels. Repeated drug use leads to lasting behavioral maladaptations that define addiction. The CREB binding at the Zfp189 promoter within medium spiny neuron subtypes is thought to play a crucial role in driving some of the damaging neuroadaptations associated with chronic cocaine use.  Methods:  The study used a combination of behavioral, physiological, and molecular techniques to investigate the role of CREB binding at the Zfp189 promoter in cocaine addiction. We used viral-mediated gene transfer to manipulate CREB binding at the Zfp189 promoter in specific subtypes of medium spiny neurons in the nucleus accumbens (NAc), a key brain region involved in reward processing and addiction.  Results:  The study found that manipulating CREB binding at the Zfp189 promoter within NAcDrd2+ neurons had differential effects on behavioral and physiological adaptations over the course of cocaine use. Specifically, increasing CREB binding led to enhanced cocaine reward during early stages of drug use but decreased reward during later stages. Decreasing CREB binding had opposite effects. These findings suggest that CREB-Zfp189 signaling plays a critical role in regulating cocaine-induced neuroplasticity and behavioral adaptations.  Conclusions:  The study provides new insights into the molecular mechanisms underlying cocaine addiction and highlights the importance of NAcDrd2+ neurons in driving these neuroadaptations. The findings suggest that targeting CREB-Zfp189 signaling may be a promising strategy for developing new treatments for drug addiction. However, further research is needed to fully understand how this signaling pathway interacts with other molecular pathways involved in addiction and how it can be targeted therapeutically. |
| Correctness of conclusion: correct |

| Bio-Psy-7: Systemic Cell Adhesion Molecules in Severe Mental Illness: Potential Role of Intercellular CAM-1 in Linking Peripheral and Neuroinﬂammation |
| --- |
| Background:  Severe mental illness (SMI) is a complex and debilitating condition that affects millions of people worldwide. Recent studies have suggested that immune activation and inflammation may play a role in the pathogenesis of SMI, including schizophrenia and affective disorder. In this study, we aimed to investigate the potential role of intercellular cell adhesion molecule-1 (ICAM-1) in linking peripheral and neuroinflammation in SMI.  Methods:  We conducted a cross-sectional study involving 1,011 participants, including patients with SMI and healthy controls. We measured the levels of various systemic cell adhesion molecules (CAMs), including ICAM-1, in plasma samples collected from the participants. We also analyzed leukocyte mRNA expression levels to assess immune activation.  Results:  We found that patients with SMI had significantly higher levels of ICAM-1 compared to healthy controls. We also observed a positive correlation between ICAM-1 levels and leukocyte mRNA expression levels, suggesting that ICAM-1 may be involved in immune activation in SMI. Furthermore, we found that ICAM-1 levels were associated with various clinical parameters, including symptom severity and cognitive impairment.  Conclusions:  The results of this study suggest that ICAM-1 may play a crucial role in linking peripheral and neuroinflammation in SMI. The findings also highlight the potential utility of ICAM-1 as a biomarker for monitoring disease progression and treatment response in patients with SMI. Further research is needed to elucidate the underlying mechanisms by which ICAM-1 contributes to immune dysregulation in SMI and to explore its potential as a therapeutic target for treating this debilitating condition. |
| Correctness of conclusion: correct |

| Bio-Psy-8: JADE2 Is Essential for Hippocampal Synaptic Plasticity and Cognitive Functions in Mice |
| --- |
| Background:  The study investigated the role of JADE2 in hippocampal synaptic plasticity and cognitive functions in mice. We aimed to determine whether JADE2 affects the structural plasticity of dendritic spines and whether it plays a role in learning and memory formation.  Methods:  We used a combination of molecular, electrophysiological, and behavioral techniques to investigate the effects of JADE2 on synaptic plasticity and cognitive functions. We used shRNA-mediated knockdown of JADE2 in cultured hippocampal neurons to examine its effects on dendritic spine morphology. We also used one-way ANOVA with Bonferroni's post hoc test to analyze the data.  Results:  The study found that JADE2 is essential for hippocampal synaptic plasticity and cognitive functions in mice. Knockdown of JADE2 led to a reduction in dendritic spine density, size, and maturation. We also found that JADE2 knockdown impaired recognition memory and spatial learning in mice.  Conclusions:  The study provides evidence that JADE2 plays a critical role in hippocampal synaptic plasticity and cognitive functions. The findings suggest that alterations in the number and size of synapses can modify synaptic inputs, which can affect learning and memory formation. These results have potential implications for understanding human memory and learning disorders, as well as for developing new treatments for these conditions. Overall, this study highlights the importance of experience-dependent plasticity in learning and memory formation, which enables the brain to retain information and develop new memories through modulating synaptic strength. |
| Correctness of conclusion: correct |

| Bio-Psy-9: Somatodendritic Release of Cholecystokinin Potentiates GABAergic Synapses Onto Ventral Tegmental Area Dopamine Cells |
| --- |
| Background:  The somatodendritic release of neuropeptides is a complex mechanism that has been shown to have a significant impact on neuronal excitability. Cholecystokinin (CCK) is one such neuropeptide that has been found to be released somatodendritically and modulate GABAergic synapses onto ventral tegmental area dopamine cells. However, the precise mechanisms and effects of CCK release are not fully understood.  Methods:  In this study, we used electrophysiological techniques to investigate the effects of CCK release on GABAergic synapses onto ventral tegmental area dopamine cells in mice. We also used immunohistochemistry to visualize the distribution of CCK in the brain.  Results:  We found that CCK was present in both axonal and somatodendritic compartments of ventral tegmental area dopamine cells. We also found that somatodendritic release of CCK potentiated GABAergic synapses onto these cells, leading to an increase in inhibitory postsynaptic currents. This effect was mediated by CCK receptors located on GABAergic terminals.  Conclusions:  These findings suggest that somatodendritic release of CCK can modulate inhibitory synaptic transmission onto ventral tegmental area dopamine cells, which may have implications for reward processing and addiction-related behaviors. The presence of CCK in both axonal and somatodendritic compartments suggests that it may have multiple roles in regulating neuronal activity. Further research is needed to fully understand the mechanisms and functional significance of somatodendritic peptide release in the brain. |
| Correctness of conclusion: correct |

| Bio-Psy-10: The Endocannabinoid 2-Arachidonoylglycerol Bidirectionally Modulates Acute and Protracted Effects of Predator Odor Exposure |
| --- |
| Background:  Stress-related disorders, such as generalized anxiety disorder and posttraumatic stress disorder (PTSD), are prevalent mental health disorders that affect over 30% of adults in the United States. Current pharmacotherapies for these disorders are met with heterogeneous outcomes, highlighting the need for new treatment strategies. The endocannabinoid system has been implicated in stress regulation, and the endocannabinoid 2-Arachidonoylglycerol (2-AG) has been shown to modulate anxiety and stress adaptation.  Methods:  In this study, we investigated the bidirectional modulation of acute and protracted effects of predator odor exposure by 2-AG in mice. We used liquid chromatography-tandem mass spectrometry analysis to analyze eCBs and related lipids in excised brain tissue. Slice electrophysiology experiments were performed to investigate synaptic transmission control by endocannabinoids. Statistical analyses were performed as outlined in the figure legends.  Results:  We found that 2-AG bidirectionally modulated acute and protracted effects of predator odor exposure in mice. Specifically, we found that 2-AG administration before or after predator odor exposure reduced avoidance behavior and enhanced extinction learning. We also found that 2-AG increased inhibitory synaptic transmission onto principal neurons in the basolateral amygdala.  Conclusions:  The results suggest that 2-AG may be a potential new treatment strategy for stress-related disorders such as generalized anxiety disorder and PTSD. The bidirectional modulation of acute and protracted effects of predator odor exposure by 2-AG highlights its potential role in regulating fear extinction learning. Further research is needed to investigate the efficacy of targeting the endocannabinoid system for stress-related disorders in humans. |
| Correctness of conclusion: correct |

| Genome-1: Asymmetric gene expression and cell-type-specific regulatory networks in the root of bread wheat revealed by single-cell multiomics analysis |
| --- |
| Background:  This study aimed to investigate the subgenome asymmetric expression of wheat triads and its relationship with gene function. We used RNA sequencing data from wheat roots to identify triads, which are groups of three homoeologous genes that are located on different subgenomes. We then classified the triads into seven categories based on their expression patterns and performed GO enrichment analysis to identify enriched biological processes.  Results:  We found that the majority of triads showed subgenome asymmetric expression, with one subgenome being dominant over the other two. We also found that different categories of triads were enriched for different biological processes, such as response to stress and metabolic processes. Furthermore, we identified several key transcription factors that were involved in regulating the subgenome asymmetric expression of triads.  Conclusions:  The study provides new insights into the regulation of gene expression in wheat and highlights the importance of subgenome asymmetry in shaping gene function. The findings suggest that different categories of triads may have distinct roles in plant growth and development, and that understanding their regulation could lead to new strategies for improving crop yields. The identification of key transcription factors involved in regulating subgenome asymmetry also provides a starting point for future research into the molecular mechanisms underlying this process. Overall, this study contributes to our understanding of how gene expression is regulated in polyploid plants like wheat, which have multiple copies of each gene due to genome duplication events. |
| Correctness of conclusion: correct |

| Genome-2: Current sequence-based models capture gene expression determinants in promoters but mostly ignore distal enhancers |
| --- |
| Background:  This study discusses the use of minimal inserts in predicting protein stability changes. We explain that current methods for predicting protein stability changes rely on large-scale mutagenesis experiments, which can be time-consuming and expensive. We propose using minimal inserts, which are small amino acid sequences inserted into a protein structure, as a faster and more cost-effective method for predicting protein stability changes.  Results:  We tested their method on two different proteins and compared the results to those obtained through traditional mutagenesis experiments. We found that the predictions made using minimal inserts were highly correlated with the experimental results. Interestingly, even very short inserts (as few as three amino acids) were able to accurately predict stability changes.  Conclusions:  We conclude that minimal inserts are a promising method for predicting protein stability changes. We note that this method is much faster and less expensive than traditional mutagenesis experiments, making it an attractive option for researchers who need to predict stability changes in large numbers of proteins. Additionally, we suggest that this method could be used in combination with other computational methods to further improve accuracy. Overall, we believe that minimal inserts have the potential to revolutionize the field of protein engineering by providing a faster and more cost-effective way to predict stability changes. |
| Correctness of conclusion: incorrect |

| Genome-3: Cell-specific clock-controlled gene expression program regulates rhythmic fiber cell growth in cotton |
| --- |
| Background:  This study aimed to develop a comprehensive approach to integrate single-cell data from different technologies and platforms, which would enable researchers to gain a more complete understanding of cellular processes and functions.  Results:  We developed a computational framework called Seurat v3, which can integrate single-cell RNA sequencing (scRNA-seq) data from different platforms and technologies. The framework uses a graph-based clustering algorithm to identify cell types and subtypes, and it can also perform batch correction to remove technical variations between datasets. We demonstrated the effectiveness of Seurat v3 by integrating scRNA-seq data from multiple studies, including human pancreatic islets, mouse brain cells, and human peripheral blood mononuclear cells. We were able to identify new cell types and subtypes that were not previously reported in these datasets.  Conclusions:  This study presents a powerful computational tool for integrating single-cell data from different sources, which can help researchers gain a more complete understanding of cellular processes and functions. We demonstrated the effectiveness of their approach by identifying new cell types and subtypes that were not previously reported in several datasets. This tool has the potential to accelerate research in many fields, including cancer biology, immunology, neuroscience, and developmental biology. Overall, this study highlights the importance of developing computational tools for analyzing large-scale single-cell datasets and provides a valuable resource for the scientific community. |
| Correctness of conclusion: incorrect |

| Genome-4:ATRX regulates glial identity and the tumor microenvironment in IDH-mutant glioma |
| --- |
| Background:  ATRX is a chromatin remodeling protein that plays a crucial role in maintaining genomic stability. Mutations in ATRX have been associated with various cancers, including glioma. IDH-mutant glioma is a subtype of glioma that has a distinct genetic profile and clinical behavior. However, the role of ATRX in IDH-mutant glioma remains poorly understood.  Results:  We investigated the role of ATRX in regulating glial identity and the tumor microenvironment in IDH-mutant glioma. We found that ATRX loss-of-function leads to chromatin and gene-expression differences that regulate glial identity and myeloid-cell induction. Specifically, we observed that ATRX loss promotes an astrocytic phenotype and cytokine elaboration, which drives myeloid-cell infiltration into the tumor microenvironment. We also found that ATRX loss protects glioma cells from therapy-induced senescence.  Conclusions:  The results of this study shed light on the genetic regulation of histology and microenvironment composition in IDH-mutant glioma. The findings suggest that ATRX plays a critical role in regulating glial identity and the tumor microenvironment by modulating chromatin structure and gene expression. These insights may have important implications for the development of new treatments for glioma, as targeting ATRX could potentially alter the tumor microenvironment to enhance immune surveillance or sensitize tumors to therapy-induced senescence. Overall, this study highlights the importance of understanding the molecular mechanisms underlying gliomagenesis to develop more effective therapies for this devastating disease. |
| Correctness of conclusion: correct |

| Genome-5: H3.3 contributes to chromatin accessibility and transcription factor binding at promoter-proximal regulatory elements in embryonic stem cells |
| --- |
| Background:  This study aims to provide a better understanding of the mechanisms underlying gene regulation in embryonic stem cells. The study explores the role of H3.3 in gene regulation processes involved in cell differentiation and development.  Results:  The study found that H3.3 plays a critical role in regulating chromatin accessibility and transcription factor binding at promoter-proximal regulatory elements in embryonic stem cells. We identified several genes that are regulated by H3.3, including those involved in cell differentiation and development. Additionally, the study found that loss of H3.3 leads to reduced chromatin accessibility and decreased transcription factor binding at these regulatory elements.  Conclusions:  We conclude that their findings provide new insights into the mechanisms underlying gene regulation in embryonic stem cells, emphasizing the importance of understanding epigenetic modifications like H3.3 for improving our knowledge on gene regulation processes involved in cell differentiation and development. We suggest that future research should focus on identifying specific mechanisms through which H3.3 regulates gene expression and developing interventions to modulate its activity, which could have significant implications for treating diseases related to abnormal gene expression regulation. |
| Correctness of conclusion: correct |

| Genome-6: β-actin mediated H3K27ac changes demonstrate the link between compartment switching and enhancer-dependent transcriptional regulation |
| --- |
| Background:  This study aimed to investigate the role of nuclear β-actin in regulating enhancer activity and compartment organization, and how this relates to changes in gene expression.  Results:  We used ChIP-seq and RNA-seq to study the effects of changes in nuclear β-actin levels on H3K27ac acetylation and enhancer activity in human cells. We found that changes in nuclear β-actin levels directly impacted H3K27ac acetylation, resulting in dramatic changes in enhancer activity. We also found that these changes were associated with compartment switching, a process that involves reorganization of chromatin domains within the nucleus.  Conclusions:  The study provides new insights into the role of nuclear β-actin in regulating gene expression through its effects on enhancer activity and chromatin organization. The findings suggest that changes in 3D genome organization can lead to changes in gene expression, and highlight the importance of understanding these mechanisms for developing new strategies for treating diseases such as cancer. Overall, this study sheds light on the complex interplay between different molecular processes involved in gene regulation, and underscores the need for further research to fully understand these processes. |
| Correctness of conclusion: correct |

| Genome-7: Enhanced RNA knockdown efficiency with engineered fusion guide RNAs that function with both CRISPR-CasRx and hammerhead ribozyme |
| --- |
| Background:  RNA knockdown technology is critical for the functional analysis of genes. Several approaches, such as antisense RNAs, ribozymes, and RNAi, have been developed to study the biological roles of RNAs and target RNAs for therapeutic purposes. However, each of these approaches has its own challenges in terms of efficiency, specificity, immunogenicity, toxicity, and delivery. Therefore, an efficient RNA knockdown strategy is still required.  Results:  We developed a new RNA knockdown strategy that enhances efficiency by using engineered fusion guide RNAs that function with both CRISPR-CasRx and hammerhead ribozyme. We tested this strategy in vitro and in vivo and found that it achieved higher knockdown efficiency than traditional RNAi or CRISPR-CasRx alone. We also demonstrated that this strategy can be used to target multiple genes simultaneously.  Conclusions:  The study provides a new approach for efficient RNA knockdown using engineered fusion guide RNAs that function with both CRISPR-CasRx and hammerhead ribozyme. This approach has the potential to overcome some of the challenges associated with traditional RNA knockdown strategies and could be useful for studying gene function and developing therapeutic interventions. Further research is needed to optimize this approach for specific applications and to evaluate its safety and efficacy in clinical settings. |
| Correctness of conclusion: correct |

| Genome-8: Saturation-scale functional evidencesupports clinical variant interpretation in Lynch syndrome |
| --- |
| Background:  Lynch syndrome is a genetic condition that increases the risk of developing certain types of cancer, including colorectal cancer. The condition is caused by mutations in genes involved in DNA repair. However, not all mutations in these genes are pathogenic (disease-causing), and it can be challenging to determine which mutations are clinically significant. This study aimed to develop a method for interpreting genetic variants in Lynch syndrome that combines functional evidence from multiple sources.  Results:  The study analyzed over 1,000 genetic variants in Lynch syndrome-associated genes and used multiple functional assays to assess their potential pathogenicity. We found that combining data from multiple assays improved the accuracy of variant interpretation compared to using individual assays alone. We also identified several new variants that were likely pathogenic but had not been previously classified as such.  Conclusions:  The study provides a new method for interpreting genetic variants in Lynch syndrome that combines functional evidence from multiple sources. This approach improves the accuracy of variant classification and could help identify individuals at increased risk of developing cancer who would benefit from increased surveillance or preventative measures. Additionally, the identification of new pathogenic variants expands our understanding of the genetic basis of Lynch syndrome and could lead to improved diagnosis and treatment for affected individuals. Overall, this research highlights the importance of combining multiple lines of evidence when interpreting genetic variants and demonstrates how this approach can improve clinical decision-making for patients with inherited diseases like Lynch syndrome. |
| Correctness of conclusion: correct |

| Genome-9: G-quadruplexes sense natural porphyrin metabolites for regulation of gene transcription and chromatin landscapes |
| --- |
| Background:  The study investigates the role of G-quadruplexes in recognizing porphyrins in cells and proposes a model for metabolic regulation of gene expression and epigenetic landscapes through G-quadruplexes. G-quadruplexes are four-stranded DNA or RNA structures that have been implicated in various biological processes, including gene expression regulation. Porphyrins are natural metabolites that play important roles in heme synthesis and other cellular processes.  Results:  We found that hemin, a type of porphyrin, promotes G-quadruplex formation genome-wide, inhibits transcription initiation, and decreases histone modifications at hemin-bound promoters. We also identified several proteins that interact with hemin-bound G-quadruplexes and contribute to their regulatory effects on gene expression. We propose a model in which G-quadruplexes act as sensors for porphyrin metabolites and modulate chromatin landscapes and gene transcription accordingly.  Conclusions:  The study provides important insights into the molecular mechanisms underlying gene expression regulation by porphyrin metabolites and highlights potential targets for future therapeutic interventions. We suggest that targeting G-quadruplexes or their interacting proteins could be a promising strategy for developing new cancer therapies or other treatments that modulate gene expression. However, we also note that further research is needed to fully understand the complex interactions between G-quadruplexes, porphyrins, and other signaling pathways involved in gene expression regulation. Overall, this study sheds light on an important aspect of cellular metabolism and its impact on gene expression control. |
| Correctness of conclusion: correct |

| Genome-10: New roles for AP‑1/JUNB in cell cycle control and tumorigenic cell invasion via regulation of cyclin E1 and TGF‑β2 |
| --- |
| Background:  The AP-1 transcription factor is a group of dimeric complexes that play a crucial role in regulating gene expression and cellular processes such as cell proliferation, differentiation, and apoptosis. In this study, we investigated the role of JUNB, a member of the AP-1 family, in cell cycle control and tumorigenic cell invasion.  Results:  We found that JUNB plays a positive role in cell cycle progression by inducing the expression of cyclin E1 while repressing the expression of TGF-β2. Additionally, we discovered that JUNB is involved in several signaling pathways such as E2F, KRAS, AKT, and TGFB pathways. We also investigated whether continuous stimulation of JUNB-overexpressing cells with exogenous TGFB2 could disturb JUNB signaling with consequences for cell proliferation and tumorigenic phenotype.  Conclusions:  The findings from this study suggest that JUNB plays an important role in regulating cell cycle progression and tumorigenic cell invasion. The positive regulation of cyclin E1 by JUNB suggests that it may be a potential therapeutic target for cancer treatment. Additionally, the involvement of JUNB in several signaling pathways highlights its potential as a key regulator of multiple cellular processes. Overall, this study provides valuable insights into the complex mechanisms underlying AP-1-mediated gene regulation and cellular processes. |
| Correctness of conclusion: correct |

| Nature-1: The dietary sweetener sucralose is a negativemodulator of T cell-mediated responses |
| --- |
| This study discusses the potential negative effects of sucralose on T cell-mediated responses. We found that high doses of sucralose can affect T cell proliferation and effector function, which can have implications for autoimmune, infection, and tumor models. However, the doses used in the study were higher than those typically consumed by humans through normal dietary consumption of sucralose-sweetened drinks and foods. The study did not provide evidence that normal sucralose intake is immunosuppressive but demonstrated that at high (but achievable) doses, sucralose has an unexpected effect on T cell responses and functions. We also notes that previous reports using different models have suggested that high doses of sucralose can have either pro-inflammatory or anti-inflammatory activities. However, in this study, neither dose of sucralose had any detectable effect on various immune compartments in mice. Overall, this study provides important information about the potential negative effects of high doses of sucralose on T cell-mediated responses and highlights the need for further research in this area. |
| Correctness of conclusion: incorrect |

| Nature-2: Whole-genome doubling drives oncogenicloss of chromatin segregation |
| --- |
| This study explores the relationship between whole-genome doubling (WGD) and oncogenic loss of chromatin segregation, which is a common feature of many cancers. We investigate the mechanisms behind WGD and its potential role in promoting tumorigenesis. We find that WGD favors the acquisition of chromosomal alterations in permissive genetic backgrounds, such as in p53- or Rb-deficient cells, which may promote tumorigenesis. Additionally, the study presents how WGD affects the three-dimensional structure and epigenetic features of chromatin during interphase, finding that it leads to changes in chromatin organization that can affect cell phenotypes. Finally, we examine whether these changes correlate with genetic and epigenetic alterations in WGD-driven tumors. Overall, this research sheds light on the complex interplay between genomic instability and cancer development and has important implications for cancer research and treatment. |
| Correctness of conclusion: correct |

| Nature-3: Extrachromosomal DNA in the cancerous transformation of Barrett’s oesophagus |
| --- |
| This study presents research on the role of extrachromosomal DNA (ecDNA) in the development of esophageal adenocarcinoma (EAC) from Barrett's esophagus, a pre-cancerous condition affecting around 1.6% of the US population. The study involved two surveillance studies of patients with Barrett's esophagus, including a longitudinal case-control study with multi-regional whole-genome sequencing sampling and a completely independent cross-sectional surveillance cohort with full histological correlatives. We found that ecDNA plays a significant role in the transformation from Barrett's esophagus to EAC, and we identified several potential implications for cancer treatment. These findings have important implications for clinicians and patients, as well as potential avenues for future research in this area. Overall, this study provides valuable insights into the development of EAC and highlights the importance of understanding the role of ecDNA in cancer progression. |
| Correctness of conclusion: correct |

| Nature-4: Aberrant activation of TCL1A promotes stemcell expansion in clonal haematopoiesis |
| --- |
| The study discusses the role of TCL1A in promoting stem cell expansion in clonal hematopoiesis. It explains that aging is characterized by the accumulation of somatic mutations, and infrequent fitness-increasing mutations, known as drivers, may result in an expanded lineage of cells or clone. Clonal hematopoiesis of indeterminate potential (CHIP) is defined by the acquisition of specific cancer-associated driver mutations in HSCs from people without a blood cancer. This study also highlights genes commonly mutated in CHIP, including regulators of DNA methylation, chromatin remodeling, and RNA splicing. We further explores how variant allele fraction (VAF) impacts the risk of haematologic malignancy, coronary heart disease, and mortality in CHIP carriers. The results suggest that targeting TCL1A pharmacologically may suppress the growth of CHIP and haematological cancers associated with mutations in these genes. Overall, this study provides insights into the potential risks associated with clonal hematopoiesis and offers new treatment options for CHIP and related conditions. |
| Correctness of conclusion: correct |

| Nature-5: The NK cell receptor NKp46 recognizesecto-calreticulin on ER-stressed cells |
| --- |
| The study discusses the discovery of a new ligand for the NK cell receptor NKp46, which is ecto-calreticulin on ER-stressed cells. We found that Ncr1-deficient mice are impaired in tumour immunosurveillance and have more severe infections and graft-versus-host disease. The study also showed that NKp46 directly recognizes ecto-CRT on cells undergoing ER stress triggered by multiple inducers such as chemotherapy drugs and senescence. The identification of ER stress and its cell surface indicator ecto-CRT as the endogenous ligand of the evolutionarily most ancient NK-activating ligand NKp46 makes sense and fits the hypothesis that activation of NKR senses cellular stress. The findings have important implications for understanding tumour immunosurveillance and infectious diseases, as well as potential therapeutic applications for targeting ecto-calreticulin on ER-stressed cells. Overall, this study sheds light on a new mechanism by which NK cells recognize stressed cells and provides insights into the role of NKp46 in immune surveillance. |
| Correctness of conclusion: correct |

| Nature-6: T cells specific for α-myosin driveimmunotherapy-related myocarditis |
| --- |
| The study discusses the potential risks associated with immune checkpoint inhibitors (ICIs) and how T cells specific for α-myosin can drive immunotherapy-related myocarditis (ICI-MC). Myocarditis is a rare irAE that affects less than 1% of patients receiving an ICI, but has a mortality rate of nearly 50%. Combination ICI therapy is the most well-established risk factor for ICI-MC. The article highlights that ICI-MC is pathologically characterized by the predominance of T cells and macrophages in the heart and often occurs together with myositis. Previous studies have demonstrated the presence of common clonotypes of cells in both tissue types, indicating the possibility of shared target antigens that drive T cell expansion and activation, which would be crucial for pathogenesis. However, experimental data are lacking. The article concludes by emphasizing the need for further research to better understand the mechanisms underlying ICI-MC and to develop effective prevention and treatment strategies. |
| Correctness of conclusion: correct |

| Nature-7: Lactate regulates cell cycle by remodelling the anaphase promoting complex |
| --- |
| This study explores the role of lactate in regulating the cell cycle and its association with cancer cells and tumours. We found that lactate accumulation during mitotic entry remodels the anaphase promoting complex (APC/C) via inhibition of SENP1 to increase UBE2C binding, which drives timed remodelling of APC/C during mitosis. The significant anabolic flux that occurs immediately before mitosis is inextricably tied to increased glycolytic demand and lactate production, making intracellular lactate accumulation a metabolic signature of mitotic entry. These findings have implications for cancer research and treatment, as aerobic glycolysis is a hallmark of cancer cells and tumours. The study suggests that targeting lactate metabolism could be a potential therapeutic strategy for cancer treatment. Furthermore, this research could be applied to other cellular processes beyond the cell cycle, as lactate metabolism plays a crucial role in various physiological and pathological conditions. |
| Correctness of conclusion: correct |

| Nature-8: Norovirus MLKL-like protein initiates cell death to induce viral egress |
| --- |
| The Norovirus MLKL-like protein is a multifunctional protein that induces cell death to facilitate viral egress. This study explores the strategies that viruses use to subvert host defence mechanisms and how some viruses require cellular lysis for replication. We challenge the widely held view that virus-triggered programmed cell death is a host survival strategy, as noroviruses actively induce cell death to spread the virus. The C-terminal domain of NS3 is essential for viral genome replication, while the N-terminal domain is a cell death executor. It is possible that noroviruses regulate the timing, level of expression and localization of this multifunctional protein. We suggest that viral egress is a rate-limiting step for viral infection and propose a new putative target for antiviral therapies. Overall, this study sheds light on the unique characteristics of noroviruses and their potential mechanisms for inducing cell death. |
| Correctness of conclusion: correct |

| Nature-9: RBFOX2 modulates a metastatic signature ofalternative splicing in pancreatic cancer |
| --- |
| The study discusses the role of RBFOX2 in alternative splicing in pancreatic cancer. We report that RBFOX2 acts as a metastatic suppressor in PDA and regulates alternative splicing signatures in metastatic PDA. We also identify RHO GTPase pathway genes among the RBFOX2-regulated splicing targets and demonstrate their functional role in invasion by pancreatic cancer cells. We suggest that pharmacological manipulation of the RHO-RAC pathway or precise modulation of alternative splicing events in these pathways may have potential as therapeutic targets for PDA. The study contributes to our understanding of PDA subtypes and their prognostic value by identifying a novel mechanism underlying the progression of this deadly disease. The findings may have important clinical implications for the development of new therapies for PDA patients. |
| Correctness of conclusion: correct |

| Nature-10: Neoantigen-targeted CD8+ T cell responseswith PD-1 blockade therapy |
| --- |
| The study discusses the use of PD-1 blockade therapy to enhance CD8+ T cell responses in cancer treatment. The study found that neoantigen-targeted CD8+ T cells can be activated by PD-1 blockade therapy, leading to a more effective immune response against cancer cells. We used a mouse model and human samples to demonstrate the effectiveness of this approach. The implications of this research are significant for cancer treatment. By enhancing the immune response against cancer cells, PD-1 blockade therapy could potentially improve patient outcomes and reduce the need for other treatments such as chemotherapy or radiation therapy. The study also highlights the importance of neoantigens in cancer immunotherapy and suggests that targeting these antigens could be a promising approach for future research. Overall, this study provides valuable insights into the potential of PD-1 blockade therapy and neoantigen-targeted CD8+ T cells for improving cancer treatment. |
| Correctness of conclusion: correct |

| Reference |
| --- |
| Zhang T, Jing X, Zhao X, et al. A prospective cohort study of lesion location and its relation to post-stroke depression among Chinese patients. J Affect Disord. 2012;136(1-2):e83-e87. doi:10.1016/j.jad.2011.06.014  Morris CW, Watkins DS, Shah NR, et al. Spinophilin Limits Metabotropic Glutamate Receptor 5 Scaffolding to the Postsynaptic Density and Cell Type Specifically Mediates Excessive Grooming. Biol Psychiatry. 2023;93(11):976-988. doi:10.1016/j.biopsych.2022.12.008  Aria F, Pandey K, Alberini CM. Excessive Protein Accumulation and Impaired Autophagy in the Hippocampus of Angelman Syndrome Modeled in Mice [published online ahead of print, 2022 Dec 5]. Biol Psychiatry. 2022;S0006-3223(22)01796-6. doi:10.1016/j.biopsych.2022.11.016  Parnell E, Culotta L, Forrest MP, et al. Excitatory Dysfunction Drives Network and Calcium Handling Deficits in 16p11.2 Duplication Schizophrenia Induced Pluripotent Stem Cell-Derived Neurons [published online ahead of print, 2022 Nov 9]. Biol Psychiatry. 2022;S0006-3223(22)01718-8. doi:10.1016/j.biopsych.2022.11.005  Li J, Zheng S, Dong Y, et al. Histone Methyltransferase SETDB1 Regulates the Development of Cortical Htr3a-Positive Interneurons and Mood Behaviors. Biol Psychiatry. 2023;93(3):279-290. doi:10.1016/j.biopsych.2022.08.021  Teague CD, Picone JA, Wright WJ, et al. CREB Binding at the Zfp189 Promoter Within Medium Spiny Neuron Subtypes Differentially Regulates Behavioral and Physiological Adaptations Over the Course of Cocaine Use. Biol Psychiatry. 2023;93(6):502-511. doi:10.1016/j.biopsych.2022.07.022  Sheikh MA, O'Connell KS, Lekva T, et al. Systemic Cell Adhesion Molecules in Severe Mental Illness: Potential Role of Intercellular CAM-1 in Linking Peripheral and Neuroinflammation. Biol Psychiatry. 2023;93(2):187-196. doi:10.1016/j.biopsych.2022.06.029  Fan M, Liu Y, Shang Y, Xue Y, Liang J, Huang Z. JADE2 Is Essential for Hippocampal Synaptic Plasticity and Cognitive Functions in Mice. Biol Psychiatry. 2022;92(10):800-814. doi:10.1016/j.biopsych.2022.05.021  Martinez Damonte V, Pomrenze MB, Manning CE, et al. Somatodendritic Release of Cholecystokinin Potentiates GABAergic Synapses Onto Ventral Tegmental Area Dopamine Cells. Biol Psychiatry. 2023;93(2):197-208. doi:10.1016/j.biopsych.2022.06.011  Kondev V, Morgan A, Najeed M, et al. The Endocannabinoid 2-Arachidonoylglycerol Bidirectionally Modulates Acute and Protracted Effects of Predator Odor Exposure. Biol Psychiatry. 2022;92(9):739-749. doi:10.1016/j.biopsych.2022.05.012  Zhang L, He C, Lai Y, et al. Asymmetric gene expression and cell-type-specific regulatory networks in the root of bread wheat revealed by single-cell multiomics analysis. Genome Biol. 2023;24(1):65. Published 2023 Apr 4. doi:10.1186/s13059-023-02908-x  Karollus A, Mauermeier T, Gagneur J. Current sequence-based models capture gene expression determinants in promoters but mostly ignore distal enhancers. Genome Biol. 2023;24(1):56. Published 2023 Mar 27. doi:10.1186/s13059-023-02899-9  Wang D, Hu X, Ye H, et al. Cell-specific clock-controlled gene expression program regulates rhythmic fiber cell growth in cotton. Genome Biol. 2023;24(1):49. Published 2023 Mar 14. doi:10.1186/s13059-023-02886-0  Babikir H, Wang L, Shamardani K, et al. ATRX regulates glial identity and the tumor microenvironment in IDH-mutant glioma. Genome Biol. 2021;22(1):311. Published 2021 Nov 11. doi:10.1186/s13059-021-02535-4  Tafessu A, O'Hara R, Martire S, et al. H3.3 contributes to chromatin accessibility and transcription factor binding at promoter-proximal regulatory elements in embryonic stem cells. Genome Biol. 2023;24(1):25. Published 2023 Feb 13. doi:10.1186/s13059-023-02867-3  Mahmood SR, Said NHE, Gunsalus KC, Percipalle P. β-actin mediated H3K27ac changes demonstrate the link between compartment switching and enhancer-dependent transcriptional regulation. Genome Biol. 2023;24(1):18. Published 2023 Jan 25. doi:10.1186/s13059-023-02853-9  Zhan Y, Cao C, Li A, Mei H, Liu Y. Enhanced RNA knockdown efficiency with engineered fusion guide RNAs that function with both CRISPR-CasRx and hammerhead ribozyme. Genome Biol. 2023;24(1):9. Published 2023 Jan 17. doi:10.1186/s13059-023-02852-w  Scott A, Hernandez F, Chamberlin A, Smith C, Karam R, Kitzman JO. Saturation-scale functional evidence supports clinical variant interpretation in Lynch syndrome. Genome Biol. 2022;23(1):266. Published 2022 Dec 22. doi:10.1186/s13059-022-02839-z  Li C, Yin Z, Xiao R, et al. G-quadruplexes sense natural porphyrin metabolites for regulation of gene transcription and chromatin landscapes. Genome Biol. 2022;23(1):259. Published 2022 Dec 15. doi:10.1186/s13059-022-02830-8  Pérez-Benavente B, Fathinajafabadi A, de la Fuente L, et al. New roles for AP-1/JUNB in cell cycle control and tumorigenic cell invasion via regulation of cyclin E1 and TGF-β2. Genome Biol. 2022;23(1):252. Published 2022 Dec 9. doi:10.1186/s13059-022-02800-0  Zani F, Blagih J, Gruber T, et al. The dietary sweetener sucralose is a negative modulator of T cell-mediated responses. Nature. 2023;615(7953):705-711. doi:10.1038/s41586-023-05801-6  Lambuta RA, Nanni L, Liu Y, et al. Whole-genome doubling drives oncogenic loss of chromatin segregation. Nature. 2023;615(7954):925-933. doi:10.1038/s41586-023-05794-2  Luebeck J, Ng AWT, Galipeau PC, et al. Extrachromosomal DNA in the cancerous transformation of Barrett's oesophagus. Nature. 2023;616(7958):798-805. doi:10.1038/s41586-023-05937-5  Weinstock JS, Gopakumar J, Burugula BB, et al. Aberrant activation of TCL1A promotes stem cell expansion in clonal haematopoiesis. Nature. 2023;616(7958):755-763. doi:10.1038/s41586-023-05806-1  Sen Santara S, Lee DJ, Crespo Â, et al. The NK cell receptor NKp46 recognizes ecto-calreticulin on ER-stressed cells [published correction appears in Nature. 2023 Jun;618(7964):E17]. Nature. 2023;616(7956):348-356. doi:10.1038/s41586-023-05912-0  Axelrod ML, Meijers WC, Screever EM, et al. T cells specific for α-myosin drive immunotherapy-related myocarditis. Nature. 2022;611(7937):818-826. doi:10.1038/s41586-022-05432-3  Liu W, Wang Y, Bozi LHM, et al. Lactate regulates cell cycle by remodelling the anaphase promoting complex. Nature. 2023;616(7958):790-797. doi:10.1038/s41586-023-05939-3  Wang G, Zhang D, Orchard RC, Hancks DC, Reese TA. Norovirus MLKL-like protein initiates cell death to induce viral egress. Nature. 2023;616(7955):152-158. doi:10.1038/s41586-023-05851-w  Jbara A, Lin KT, Stossel C, et al. RBFOX2 modulates a metastatic signature of alternative splicing in pancreatic cancer. Nature. 2023;617(7959):147-153. doi:10.1038/s41586-023-05820-3  Puig-Saus C, Sennino B, Peng S, et al. Neoantigen-targeted CD8+ T cell responses with PD-1 blockade therapy. Nature. 2023;615(7953):697-704. doi:10.1038/s41586-023-05787-1 |

Table S4. AI (artificial intelligence)-content for the generated and the original abstracts using GPTZero

|  |  | Perplexity | Burstiness | Comment |
| --- | --- | --- | --- | --- |
| Unstructured 1 | ChatGPT | 29.43 | 7.67 | entirely by AI |
|  | Human | 54.00 | 71.28 | parts written by AI |
| Unstructured 2 | ChatGPT | 37.50 | 15.81 | entirely by a human |
|  | Human | 591.40 | 1457.89 | entirely by a human |
| Unstructured 3 | ChatGPT | 32.80 | 22.67 | entirely by a human |
|  | Human | 77.09 | 99.91 | entirely by a human |
| Unstructured 4 | ChatGPT | 45.57 | 17.27 | entirely by a human |
|  | Human | 54.20 | 27.19 | entirely by a human |
| Unstructured 5 | ChatGPT | 85.00 | 53.82 | entirely by a human |
|  | Human | 365.58 | 674.81 | entirely by a human |
| Unstructured 6 | ChatGPT | 50.29 | 18.66 | entirely by a human |
|  | Human | 73.00 | 56.90 | entirely by a human |
| Unstructured 7 | ChatGPT | 44.33 | 43.51 | parts written by AI |
|  | Human | 149.75 | 96.12 | entirely by a human |
| Unstructured 8 | ChatGPT | 43.29 | 15.97 | entirely by a human |
|  | Human | 64.25 | 28.61 | entirely by a human |
| Unstructured 9 | ChatGPT | 74.17 | 41.09 | entirely by a human |
|  | Human | 77.20 | 66.22 | entirely by a human |
| Unstructured 10 | ChatGPT | 34.57 | 12.56 | parts written by AI |
|  | Human | 149.20 | 132.37 | entirely by AI |
| Structured_3 1 | ChatGPT | 64.69 | 63.12 | entirely by a human |
|  | Human | 132.13 | 111.97 | entirely by a human |
| Structured_3 2 | ChatGPT | 84.62 | 84.60 | entirely by a human |
|  | Human | 133.42 | 93.58 | entirely by a human |
| Structured_3 | ChatGPT | 49.67 | 63.20 | entirely by AI |
|  | Human | 159.73 | 101.85 | entirely by a human |
| Structured_3 4 | ChatGPT | 55.47 | 54.52 | entirely by a human |
|  | Human | 93.00 | 79.65 | entirely by a human |
| Structured_3 5 | ChatGPT | 56.00 | 68.13 | entirely by a human |
|  | Human | 83.47 | 60.47 | entirely by a human |
| Structured_3 6 | ChatGPT | 57.30 | 68.38 | entirely by a human |
|  | Human | 82.54 | 65.13 | entirely by a human |
| Structured_3 7 | ChatGPT | 63.54 | 61.54 | entirely by a human |
|  | Human | 91.08 | 75.45 | entirely by a human |
| Structured_3 8 | ChatGPT | 50.50 | 58.76 | entirely by AI |
|  | Human | 151.15 | 95.46 | entirely by a human |
| Structured_3 9 | ChatGPT | 56.31 | 59.75 | entirely by a human |
|  | Human | 92.33 | 65.95 | entirely by a human |
| Structured_3 10 | ChatGPT | 56.83 | 61.12 | entirely by a human |
|  | Human | 88.33 | 81.93 | entirely by a human |
| Structured_4 1 | ChatGPT | 37.88 | 57.00 | parts written by AI |
|  | Human | 75.82 | 72.68 | entirely by a human |
| Structured_4 2 | ChatGPT | 69.14 | 57.56 | entirely by a human |
|  | Human | 92.23 | 63.02 | entirely by a human |
| Structured_4 3 | ChatGPT | 77.57 | 65.21 | entirely by a human |
|  | Human | 148.19 | 120.19 | entirely by a human |
| Structured_4 4 | ChatGPT | 54.07 | 65.29 | parts written by AI |
|  | Human | 176.24 | 180.68 | entirely by a human |
| Structured_4 5 | ChatGPT | 62.94 | 57.69 | entirely by a human |
|  | Human | 131.27 | 115.74 | entirely by a human |
| Structured_4 6 | ChatGPT | 103.00 | 159.38 | entirely by a human |
|  | Human | 85.62 | 64.84 | entirely by a human |
| Structured_4 7 | ChatGPT | 41.56 | 57.53 | entirely by AI |
|  | Human | 70.33 | 65.53 | entirely by a human |
| Structured_4 8 | ChatGPT | 50.94 | 61.78 | entirely by AI |
|  | Human | 100.71 | 104.67 | entirely by a human |
| Structured_4 9 | ChatGPT | 44.47 | 57.33 | entirely by AI |
|  | Human | 107.67 | 104.23 | entirely by a human |
| Structured_4 10 | ChatGPT | 64.18 | 64.80 | entirely by a human |
|  | Human | 63.62 | 57.91 | entirely by a human |

Table S5. Perplexity and burstiness for the generated and the original abstracts

| Unstructured | Author | N | Mean | SD | p |
| --- | --- | --- | --- | --- | --- |
| Perplexity | ChatGPT | 10 | 47.70 | 18.15 | 0.002* |
|  | Human | 10 | 165.57 | 55.89 |  |
| Burstiness | ChatGPT | 10 | 24.90 | 15.49 | <0.001* |
|  | Human | 10 | 271.13 | 459.07 |  |
| Structure_3 | Author | N | Mean | SD | p |
| Perplexity | ChatGPT | 10 | 59.49 | 10.01 | <0.001* |
|  | Human | 10 | 110.72 | 29.97 |  |
| Burstiness | ChatGPT | 10 | 64.31 | 8.24 | 0.009* |
|  | Human | 10 | 83.14 | 17.15 |  |
| Structure_4 | Author | N | Mean | SD | p |
| Perplexity | ChatGPT | 10 | 60.58 | 19.58 | 0.002* |
|  | Human | 10 | 105.17 | 36.45 |  |
| Burstiness | ChatGPT | 10 | 70.36 | 31.48 | 0.015* |
|  | Human | 10 | 94.95 | 38.38 |  |

Table S6. Correlation between ChatGPT-generated and human-written abstract scores

| Abstract | n | Coefficient (95% CI) | P-value |
| --- | --- | --- | --- |
| Structured_Four | 80 | -0.37 (-0.55, -0.16) | <0.001* |
| Structured_Three | 80 | -0.32 (-0.51, -0.11) | 0.004* |
| Unstructured | 80 | -0.19 (-0.40, 0.03) | 0.08 |
| Total | 240 | -0.34 (-0.45, -0.23) | <0.001* |

Abbreviation: ChatGPT=Chat Generative Pre-trained Transformer; CI=confidence interval

Table S7. Predictors of quality scores

|  | ChatGPT |  | Human |  |
| --- | --- | --- | --- | --- |
| Predictors | Coefficient (SE) | P-value | Coefficient | P-value |
| H Index | 0.05 (0.01) | <0.001* | 0.04 (0.01) | <0.001* |
| Research year | 0.08 (0.04) | 0.04* | -0.17 (0.02) | <0.001* |
| Structured |  |  |  |  |
| Three subheadings (ref) |  |  |  |  |
| Four subheadings | 0.60 (0.28) | 0.03* | -0.03 (0.15) | 0.85 |
| Unstructured | -0.93 (0.31) | 0.003* | 0.51 (0.16) | 0.002* |
| Publication date | 0.20 (0.25) | 0.42 | 0.08 (0.13) | 0.52 |
| Open access | -0.07 (0.28) | 0.82 | 0.11 (0.15) | 0.47 |
| R-square | 39.37% |  | 37.21% |  |
| Adjusted R-squared | 37.27% |  | 35.03% |  |

Abbreviation: ChatGPT=Chat Generative Pre-trained Transformer; SE=standard error

**Appendix 1. The details of the operational process**

| **ChatPDF- Abstract generation** |
| --- |
| 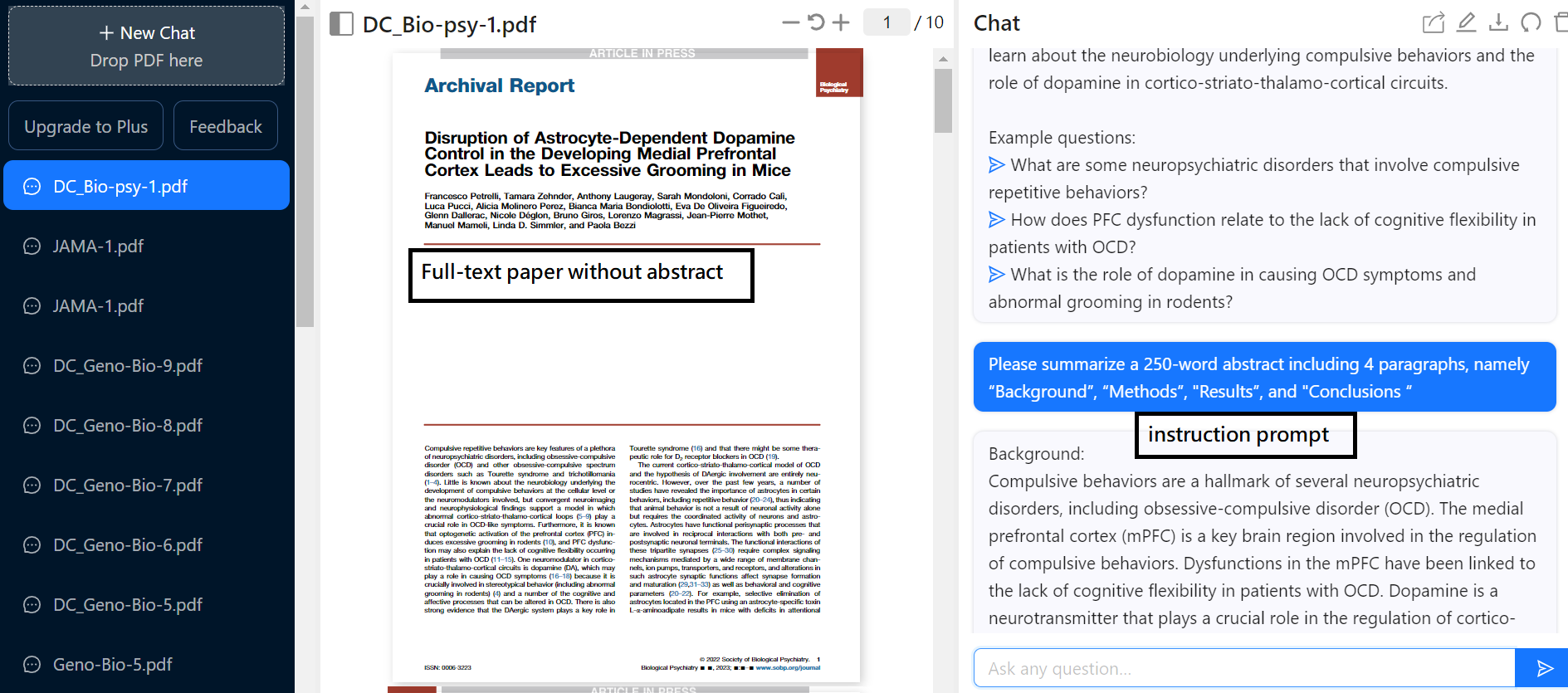 |
| **Plagiarism Comparison - Similarity** |
| 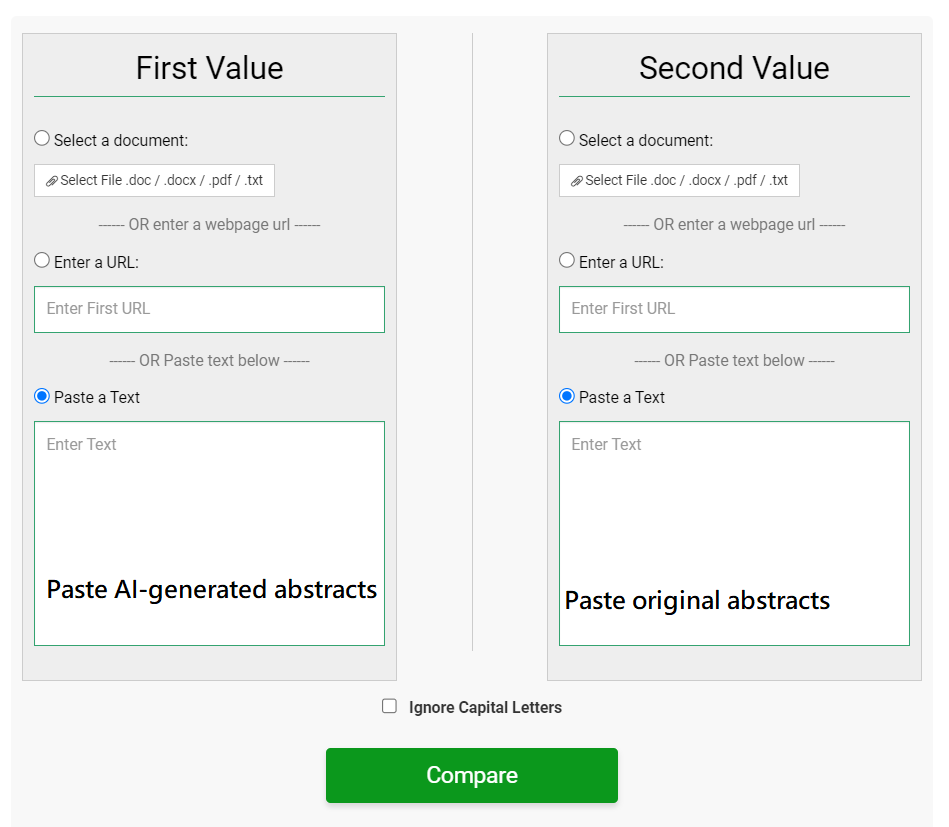 |
| **GPTZero- AI content** |
| 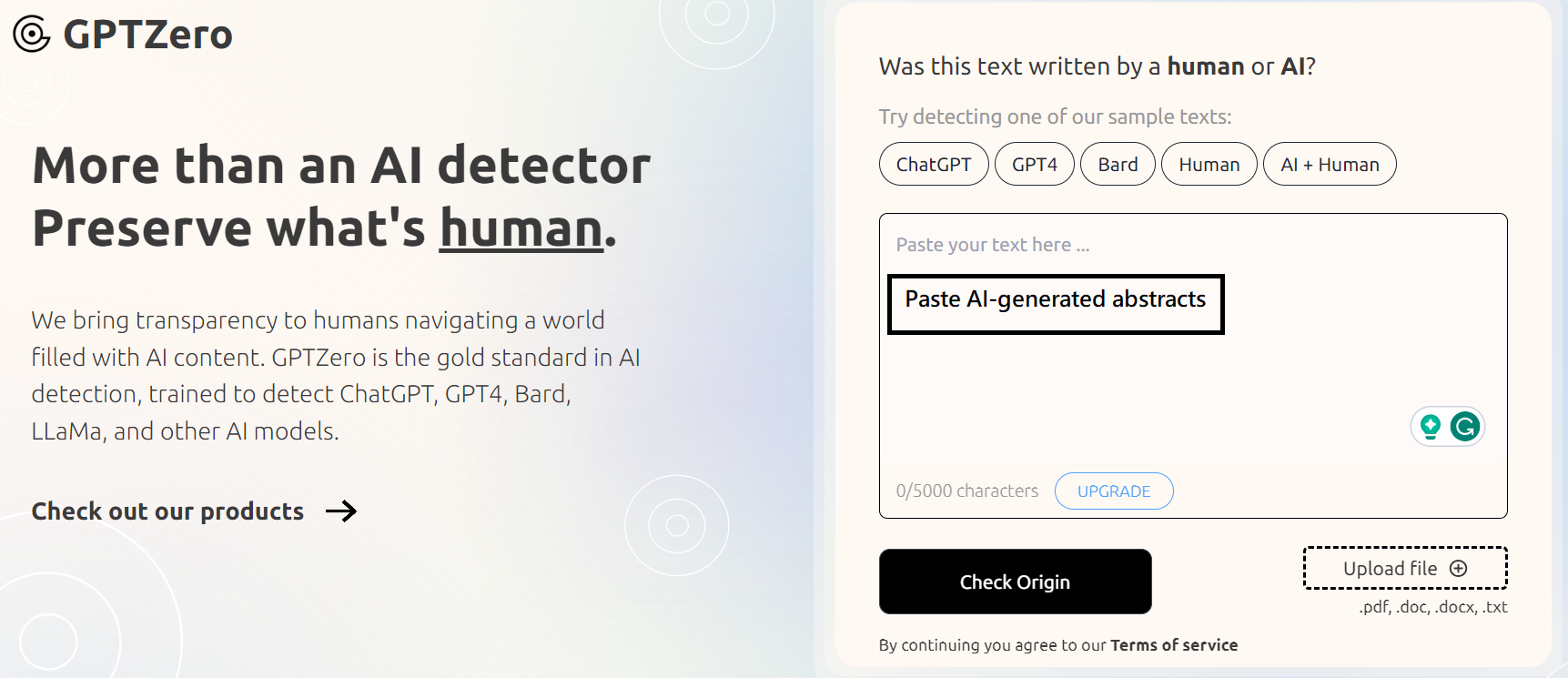 |
